# Autotoxicity mechanism of *Oryza sativa*: transcriptome response in rice roots exposed to ferulic acid

**DOI:** 10.1186/1471-2164-14-351

**Published:** 2013-05-25

**Authors:** Wen-Chang Chi, Yun-An Chen, Yu-Chywan Hsiung, Shih-Feng Fu, Chang-Hung Chou, Ngoc Nam Trinh, Ying-Chih Chen, Hao-Jen Huang

**Affiliations:** 1 Department of Life Sciences, National Cheng Kung University, No. 1 University Rd. 701, Tainan, Taiwan, ROC; 2 Department of Biological Sciences, National Sun Yat-Sen University, No. 70, Lienhai Rd. 80424, Kaohsiung, Taiwan, ROC; 3 Department of Biology, National Changhua University of Education, No. 1, Jin-De Road, Changhua City 500, Taiwan, ROC; 4 Graduate Institute of Ecology and Evolutionary Biology, College of Life Sciences, China Medical University, 91, Hsueh-Shih Road, Taichung 404, Taiwan, ROC

**Keywords:** Allelochemical, Ferulic acid, Microarray, Protein kinase, Rice, Autotoxicity

## Abstract

**Background:**

Autotoxicity plays an important role in regulating crop yield and quality. To help characterize the autotoxicity mechanism of rice, we performed a large-scale, transcriptomic analysis of the rice root response to ferulic acid, an autotoxin from rice straw.

**Results:**

Root growth rate was decreased and reactive oxygen species, calcium content and lipoxygenase activity were increased with increasing ferulic acid concentration in roots. Transcriptome analysis revealed more transcripts responsive to short ferulic-acid exposure (1- and 3-h treatments, 1,204 genes) than long exposure (24 h, 176 genes). Induced genes were involved in cell wall formation, chemical detoxification, secondary metabolism, signal transduction, and abiotic stress response. Genes associated with signaling and biosynthesis for ethylene and jasmonic acid were upregulated with ferulic acid. Ferulic acid upregulated ATP-binding cassette and amino acid/auxin permease transporters as well as genes encoding signaling components such as leucine-rich repeat VIII and receptor-like cytoplasmic kinases VII protein kinases, APETALA2/ethylene response factor, WRKY, MYB and Zinc-finger protein expressed in inflorescence meristem transcription factors.

**Conclusions:**

The results of a transcriptome analysis suggest the molecular mechanisms of plants in response to FA, including toxicity, detoxicification and signaling machinery. FA may have a significant effect on inhibiting rice root elongation through modulating ET and JA hormone homeostasis. FA-induced gene expression of AAAP transporters may contribute to detoxicification of the autotoxin. Moreover, the WRKY and Myb TFs and LRR-VIII and SD-2b kinases might regulate downstream genes under FA stress but not general allelochemical stress. This comprehensive description of gene expression information could greatly facilitate our understanding of the mechanisms of autotoxicity in plants.

## Background

Monoculture of crops leads to decreased growth and yield in the next season, with autotoxicity the major culprit [1-3]. Autotoxicity occurs when a plant releases toxic chemical substances into the environment that inhibit germination and growth of conspecific plants [4]. Recently, an increasing number of reports have provided evidence for the role of autotoxicity in replant failure and soil sickness [1]. Autotoxicity is a common problem in continuous monocropping of rice [2] because decomposing rice straw is left in fallow fields [5]. A range of secondary metabolites in rice straws, such as phenolic acids [6] and a few flavones and terpenoids [7], are potent autotoxins.

Phenolic compounds are common in soils. Whitehead [8] reported that the concentration of phenolic compounds in rhizosphere soil solution may reach 90 ppm. Various phenolic compounds such as ferulic acid (FA), o-hydroxy phenyl acetic acid, and p-coumaric acid have been isolated from decomposing rice residues in soil [5]. These compounds inhibit the growth of rice seedlings in the order of FA > p-coumaric acid > o-hydroxy phenyl acetic acid [9]. Exposure of plant roots to FA reduces water use [10], inhibits foliar expansion [11] and root elongation [12], and decreases nutrient uptake [13-15]. Further, FA exposure rapidly depolarizes root cell membranes, causing a generalized increase in membrane permeability, inducing lipid peroxidation and affecting certain enzymatic activities [16-18]. Ferulic acid may be esterified with cell wall polysaccharides, be incorporated into lignin structures, or form bridges that connect lignin with wall polysaccharides, thus resulting in cell wall rigidity and restriction of cell growth [19,20]. Ferulic acid affects cell wall-bound peroxidase (POD) and phenylalanine ammonia-lyase (PAL) activities, lignin content, and root growth in seedlings [21].

Several reports demonstrated that autotoxins induce oxidative stress in plants [22,23]. Reactive oxygen species (ROS) play a vital role in the plant defense against stresses and in cell growth and development [24,25]. Low concentrations of ROS, as a signal, can lead to repair of cellular damage, but high levels can lead to programmed cell death [26]. Calcium is a crucial regulator of growth and development in plants [27]. ROS-activated calcium channel activity is required during the growth of cells in the elongation zone of the root [28].

Both allelopathy and autotoxicity play important roles in regulating plant biodiversity and productivity [3]. Autotoxins can impact many physiological and biochemical reactions in plants such as rice, alfalfa, cucumber, tomato, corn, wheat, sugarcane [1,23]. The potential mechanisms underlying autotoxicity have been explored in alfalfa and cucumber [22,29]. In alfafa, cinnamic acid is a phenolic acid and the major autotoxin in leaves and root exudates [30]. In cucumber, autotoxins can inhibit the membrane H^+^-ATPase activity that drives the uptake of essential ions, other solutes and water [22]. However, our knowledge of an autotoxicity mechanism is poorly understood. Transcriptional profiling experiments using microarrays are being conducted to examine the effects of natural phytotoxins on the plant transcriptome [31]. Microarray analyses were used to analyze gene expression profiles of plants exposed to the allelochemicals 2(3H)-benzoxazolinone [32], fagomine, gallic acid, rutin [33], 3-(3',4'-dihydroxyphenyl)-L-alanine [34], and juglone [35].

Rice (*Oryza sativa* L.) is a model for genomic research into the responses of monocot species to environmental stresses. In this study, we used FA as a rice-model autotoxin and used microarray assay to assess alterations in rice root gene expression induced by the autotoxin. We discuss the possible involvement of reactive oxygen species (ROS) and calcium in allelochemical signal transduction pathways. These data significantly expand on previous studies examining plant transcriptional responses to allelochemicals and provides a foundation for elucidating the autotoxicity mechanism of *O. sativa*, particularly the phytotoxic effect of decomposing rice residues in soil.

## Results

### Effect of FA on growth and root architecture of rice

**Figure 1:**
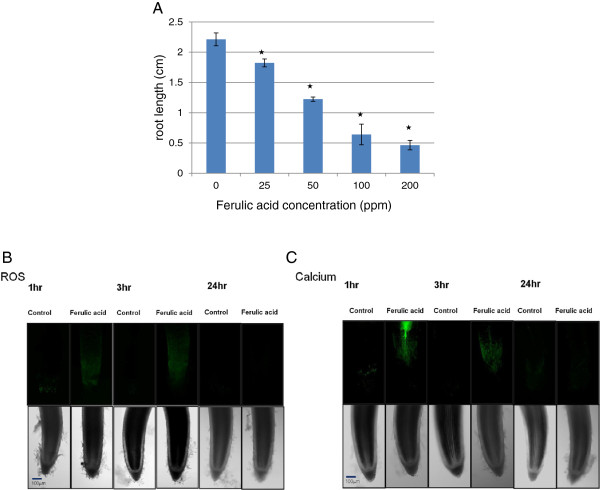
**Ferulic acid (FA) stress inhibits root elongation of rice seedlings.** (**A**) Rice roots were measured after 3 d of treatment with different concentrations of FA (0, 25, 50, 100, or 200 ppm). Results represent the means ± SD (n = 30) of 3 independent experiments. Asterisks indicate significant differences (*P* < 0.05) from the control treatment. (**B**) To assess reactive oxygen species (ROS) production under FA stress, root samples were labeled with 10 μM CM-H_2_DCF-DA for 30 min and treated with 50 ppm FA for 1–3 h. Green fluorescence indicates the presence of ROS. (**C**) To evaluate calcium accumulation under FA stress, root samples were labeled with 10 μM Oregon Green 488 BAPTA-1, a calcium indicator, for 30 min and treated with 50 ppm FA for 1–3 h. Green fluorescence indicates the presence of calcium. Five control and 5 treated roots showed similar results. Magnification for representing images was × 100.

To select an appropriate concentration of FA for stress treatments, we conducted a dose-response analysis of rice root growth 3 days after FA treatment (Figure 1A). Compared with the control, 25 ppm FA significantly reduced root growth. With 50 ppm FA, root growth was about half of the control growth, and with 200 ppm, growth was almost completely inhibited.

Compared with the control, 50 ppm FA inhibited crown root, lateral root and root hair formation. Both the number and length of lateral roots and root hairs were significantly reduced with 50 ppm FA as compared with the control (Table 1).

**Table 1:** Effect of ferulic acid treatment on number and length of crown root, lateral root and root hairs in rice

**Character **^**a**^	**Water**	**50 ppm FA**
Crown root number	6.93 ± 0.88	2.93 ± 0.70
Lateral root number/seedling	20.73 ± 2.63	6.67 ± 1.54
Lateral root length (mm)	6.15 ± 0.93	2.45 ± 0.60
Root hair number/mm^b^	73.5 ± 6.50	40.1 ± 3.07
Root hair length (μm) ^c^	599.05 ± 30.34	205.55 ± 27.29

^a^ Number and length of root hairs on seminal root measured after 24 h FA treatment; other characters were determined on seedlings after 3-day FA treatment. Data are mean ± SD.

^b^ Number of root hairs from one side of 1-mm sections at the root hair zone (3–4 mm behind the root tip) on seminal roots. Data are mean of 10 seedlings.

^c^ Length of the 20 longest root hairs from the root hair zone of each seminal root. Data represent the mean of 10 seedlings.

### Ferulic acid rapidly induced ROS and calcium accumulation in rice roots

To determine whether FA treatment induced ROS production, we labeled roots with the ROS-sensitive dye CM-H_2_DCFDA (Figure 1B) or nitroblue tetrazolium (Additional file 1: Figure S1), then treated them with 50 ppm FA for 1 or 3 h. Ferulic-acid stress significantly increased the levels of dihydrodichlorofluorescein (DCF), and thus ROS, in roots (Figure 1B, Additional file 2: Figure S2). To determine whether FA treatment induced calcium accumulation, we used a calcium indicator, Oregon green 488 BAPTA-1, before FA treatment. Calcium level was significantly increased in root tip regions with 50 ppm FA treatment for 1 or 3 h (Figure 1C and Additional file 2: Figure S2).

### Effect of FA on lipid peroxidation

Ferulic-acid–induced oxidative damage of roots was positively confirmed by Schiff’s staining in the meristem and elongation zone of roots (Additional file 3: Figure S3). Ferulic-acid–induced root oxidative damage was measured by LOX activity with non-denaturing PAGE. We detected 3 LOX isozymes in rice roots treated with 50 ppm FA for 3, 6, 12, and 24 h (Additional file 3: Figure S3).

### Expression profiling by microarray assay

To identify genes and biological pathways associated with FA toxicity and tolerance in rice roots, we used large-scale expression profiling. RNA samples were collected from root tips early (1 and 3 h) after FA treatment to examine rapid changes in global patterns of gene expression. We pooled RNA isolated from the two short (1 and 3 h) FA exposures to maximize gene discovery. Mechanisms of adaptation after long-term (24 h) FA exposure are important, but the physiological and metabolic parameters measured after long treatment periods might be distorted by the severe toxic effects of FA. We aimed to understand the primary response to FA exposure as opposed to responses to nonspecific cellular damage.

We performed microarray assays with RNA extracted from roots treated with 50 ppm FA after short (pooled from 1- and 3-h treatments) and long (24 h) exposure. This FA level is comparable to that found in rice-field soils [8,36]. In all, 1,204 genes were responsive to short FA exposure and 176 to long exposure. After short FA treatment, 972 genes were upregulated (FDR < 0.1, fold change ≥ 2) and 232 were downregulated (FDR < 0.1, fold change ≤ 0.5) (Additional file 4: Table S1).

We used GO analysis [37] to determine the functions of the 972 upregulated genes (Table 2, Additional file 5: Table S2). The most significantly enriched GO term was “response to stress” (GO:0006950, FDR 2.00E-47). Other enriched terms were “phenylpropanoid metabolic process” (GO:0009698, FDR 2.10E-07), “transmembrane transport” (GO:0055085, FDR 1.10E-12), “proteolysis” (GO:0006508, FDR 1.30E-14), “cell wall macromolecule metabolic process” (GO:0044036, FDR 6.10E-13) and “signal transduction” (GO:0007165, FDR 6.30E-05). For molecular function, the significant GO terms were “kinase activity” (GO:0016301, 1.10E-32), “calcium ion binding” (GO:0005509, FDR 7.80E-23), “transcription factor activity” (GO:0003700, FDR 9.00E-19), and “chitinase activity” (GO:0004568, FDR 1.00E-09).

**Table 2:** Gene ontology analysis of 972 up-regulated genes

**GO ID**	**GO term**	**Query item**	**Background item**	**FDR p-value**
	**biological process**			
**Regulation of biological process**				
***regulation of metabolic process***				
GO:0080090	regulation of primary metabolic process	53	324	8.30E-30
GO:0045449	regulation of transcription	51	321	3.00E-28
GO:0060255	regulation of macromolecule metabolic process	53	326	1.10E-29
GO:0010556	regulation of macromolecule biosynthetic process	52	322	4.40E-29
GO:0010468	regulation of gene expression	52	324	5.50E-29
GO:0009889	regulation of biosynthetic process	52	322	4.40E-29
GO:0051171	regulation of nitrogen compound metabolic process	51	323	3.90E-28
***regulation of cellular process***				
GO:0007165	signal transduction	11	106	6.30E-05
GO:0007242	intracellular signaling cascade	10	68	6.90E-06
**Biological regulation**				
GO:0065008	regulation of biological quality	12	14	4.40E-18
**Multi-organism process**				
GO:0051707	response to other organism	9	39	4.80E-07
GO:0009617	response to bacterium	6	7	3.80E-09
**Cellular process**				
***cellular response to stimulus***				
GO:0070887	cellular response to chemical stimulus	7	43	0.00013
GO:0055085	transmembrane transport	9	12	1.10E-12
**Metabolic process**				
***primary metabolic process***				
GO:0005975	carbohydrate metabolic process	37	138	3.60E-29
GO:0005976	polysaccharide metabolic process	12	57	1.10E-08
GO:0006022	aminoglycan metabolic process	6	21	1.70E-05
GO:0006030	chitin metabolic process	6	21	1.70E-05
GO:0016052	carbohydrate catabolic process	17	45	1.10E-16
GO:0006629	lipid metabolic process	21	81	1.20E-16
GO:0019538	protein metabolic process	80	487	4.30E-44
GO:0006508	proteolysis	23	126	1.30E-14
***secondary metabolic process***				
GO:0006721	terpenoid metabolic process	5	54	0.025
GO:0016101	diterpenoid metabolic process	5	35	0.0036
GO:0009698	phenylpropanoid metabolic process	6	11	2.10E-07
***macromolecule metabolic process***				
GO:0019538	protein metabolic process	80	487	4.30E-44
GO:0043412	macromolecule modification	50	265	3.60E-31
GO:0006464	protein modification process	50	264	3.10E-31
GO:0044036	cell wall macromolecule metabolic process	11	21	6.10E-13
GO:0016998	cell wall macromolecule catabolic process	7	21	8.60E-07
GO:0010467	gene expression	55	419	3.50E-26
GO:0009059	macromolecule biosynthetic process	56	569	1.30E-20
**Establishment of localization**				
***transport***				
GO:0006811	ion transport	13	66	5.40E-09
GO:0006812	cation transport	10	65	4.60E-06
GO:0030001	metal ion transport	10	36	1.30E-08
**Response to stimulus**				
GO:0009719	response to endogenous stimulus	8	106	0.0075
GO:0009628	response to abiotic stimulus	7	41	9.70E-05
GO:0009607	response to biotic stimulus	13	39	4.10E-12
GO:0006950	response to stress	46	103	2.00E-47
GO:0006952	defense response	16	59	3.40E-13
GO:0006979	response to oxidative stress	11	17	2.40E-14
GO:0042221	response to chemical stimulus	27	133	3.00E-18
GO:0010033	response to organic substance	8	106	0.0075
	**molecular function**			
**Molecular transducer activity**				
GO:0004871	signal transducer activity	13	32	2.00E-13
**Transporter activity**				
***substrate-specific transporter activity***				
GO:0022891	substrate-specific transmembrane transporter	14	79	4.90E-09
GO:0015075	ion transmembrane transporter activity	9	68	5.20E-05
GO:0008324	cation transmembrane transporter activity	5	62	0.04
***transmembrane transporter activity***				
GO:0016820	hydrolase activity, acting on acid anhydrides, catalyzing transmembrane movement of substances	5	17	9.30E-05
GO:0042626	ATPase activity, coupled to transmembrane movement of substances	5	17	9.30E-05
***active transmembrane transporter activity***				
GO:0015291	secondary active transmembrane transporter	7	24	2.20E-06
GO:0015399	primary active transmembrane transporter activity	5	25	0.00064
**Antioxidant activity**				
GO:0004601	peroxidase activity	8	68	0.00036
**Transcription regulator activity**				
GO:0003700	transcription factor activity	26	116	9.00E-19
**Catalytic activity**				
***oxidoreductase activity***				
GO:0004497	monooxygenase activity	21	47	2.40E-22
GO:0051213	dioxygenase activity	7	7	1.10E-11
GO:0015036	disulfide oxidoreductase activity	7	10	1.20E-09
***transferase activity***				
GO:0016757	transferase activity, transferring glycosyl groups	24	31	1.00E-33
GO:0016758	transferase activity, transferring hexosyl groups	19	30	3.70E-24
GO:0016772	transferase activity, transferring phosphorus-containing groups	54	426	5.10E-25
GO:0016773	phosphotransferase activity, alcohol group as	47	244	6.30E-30
GO:0004672	protein kinase activity	42	235	1.40E-25
GO:0016301	kinase activity	51	261	1.10E-32
***hydrolase activity***				
GO:0016798	hydrolase activity, acting on glycosyl bonds	25	87	6.40E-21
GO:0004553	hydrolase activity, hydrolyzing O-glycosy	24	85	6.50E-20
GO:0004568	chitinase activity	9	21	1.00E-09
**Binding**				
***carbohydrate binding***				
GO:0005529	sugar binding	6	10	9.10E-08
***nucleic acid binding***				
***ion binding***				
GO:0043169	cation binding	126	175	6.50E-166
GO:0046872	metal ion binding	111	173	1.60E-137
GO:0046914	transition metal ion binding	80	132	9.80E-97
GO:0008270	zinc ion binding	30	89	3.60E-27
GO:0005507	copper ion binding	6	19	9.00E-06
GO:0005509	calcium ion binding	20	39	7.80E-23

**Figure 2:**
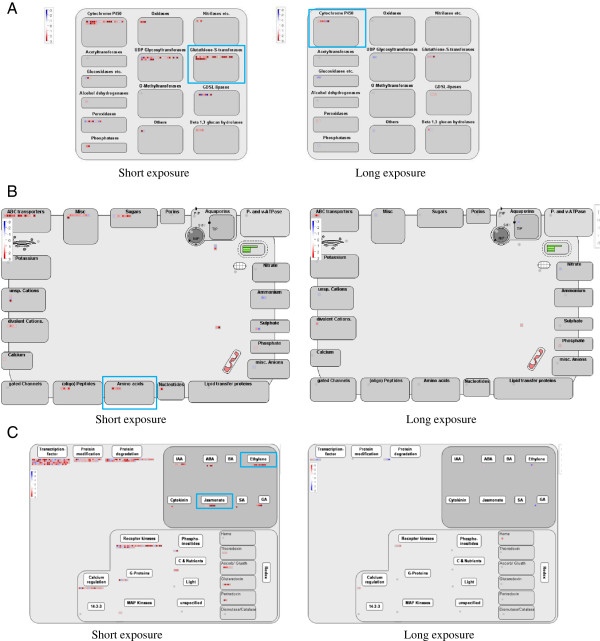
**Genes up- or down-regulated by FA stress.** MapMan was used to visualize the detoxification enzyme (**A**), transporter (**B**), and phytohormone (**C**) genes. Each BIN or subBIN is represented as a block, within which upregulated transcripts are displayed as red squares and downregulated transcripts as blue squares. Functional bins identified by the Wilcoxon rank sum statistic as being significantly changed by FA are outlined in blue.

These observations were further supported by comparison of metabolism genes with use of MapMan. The genes encoding enzymes related to detoxification were cytochrome P450, UDP glycosyltransferases, and glutathione-S-transferases (Figure 2A). RT-PCR validated the microarray findings (Additional file 6: Figure S4).

### Expression profiles of root architecture-related genes

To investigate the involvement of root architecture related genes in FA-induced stress, we analyzed the global expression profiles of genes related to 3 such gene families (Table 3). In total, 3 of the 18 root architecture related genes were slightly downregulated by FA (FDR < 0.1). FA repressed the expression of two lateral-root genes (*ARF-16*, Os06g0196700, downregulated 1.7-fold; *OsCel9C*, Os05g0212300, downregulated 1.6-fold) and one root-hair–related gene (*OsCSLD1*, Os10g0578200, downregulated 1.5-fold) in rice roots (Table 3).

**Table 3:** List of rice genes associated with crown root, lateral root, root hair formation after FA exposures

**Gene name**	**RAB-DB Locus ID**	**TIGR Locus ID**	**Short exposures**	**Long exposures**	**Description**
			**Fold change **^a^	**Fold change **^**a**^	
**Crown root-related genes**					
*OsCRL1*	Os03g0149000	LOC_Os03g05500	−1.09	1.15	Protein of unknown function DUF260 domain containing protein.
*OsCRL4*	Os03g0666100	LOC_Os03g46330	−1.21	−1.04	SEC7-like domain containing protein.
*OsCRL5*	Os07g0124700	LOC_Os07g03250	1.64	1.06	ANT (Ovule development protein aintegumenta).
*OsARL1*	Os03g0149100	LOC_Os03g05510			
*WOX11*	Os07g0684900	LOC_Os07g48560	1.86	−1.39	Homeobox domain containing protein.
**Lateral root-related genes**					
*ARF-16*	Os06g0196700	LOC_Os06g09660	−1.73*	−1.43*	Auxin response factor 1.
*AUX/IAA-1*	Os01g0178500	LOC_Os01g08320	1.88*	−1.20	AUX/IAA protein family protein.
*AUX/IAA-11*	Os03g0633500	LOC_Os03g43400	1.07	−1.40	Auxin-responsive protein IAA17 (Indoleacetic acid-induced protein 17) (Auxin response 3).
*AUX/IAA-13*	Os03g0742900	LOC_Os03g53150	1.22	−1.04	AUX/IAA protein family protein.
*AUX/IAA-23*	Os06g0597000	LOC_Os06g39590	1.77*	−1.07	Auxin-responsive protein IAA14 (Indoleacetic acid-induced protein 14) (SOLITARY-ROOT protein).
*AUX/IAA-29*	Os12g0601400	LOC_Os12g40900	1.16*	−1.28	Auxin-responsive protein (Aux/IAA) (Fragment).
*OsCel9C*	Os05g0212300	LOC_Os05g12150	−1.59*	1.43*	Endo-beta-1,4-glucanase precursor (EC 3.2.1.4).
**Root hair-related genes**					
*OsEXPA30*	Os10g0535900	LOC_Os10g39110			
*OsRHL1*	Os06g0184000	LOC_Os06g08500			
*OsCSLD1*	Os10g0578200	LOC_Os10g42750	1.47	−1.94*	Cellulose synthase-9.
*OsEXPA17*	Os06g0108600	LOC_Os06g01920			
*OsEXPB5*	Os04g0552200	LOC_Os04g46650	1.22	1.11	Beta-expansin 5.
*OsAPY1*	Os07g0682800	LOC_Os07g48430	1.35	−1.05	Apyrase.

^a^ The fold change in expression of each gene after FA treatment was calculated by the mean from 3 biological replicates and false discovery rate <0.1 is shown with a asterisk.

### Polysaccharide and cell wall metabolism

To investigate the involvement of cell-wall–related genes in FA-induced stress, we analyzed the global expression profiles of genes related to 34 such gene families (Additional file 7: Table S3). In total, 30 of the 639 cell-wall–related genes showed significant responses to FA: 16 were upregulated and 14 downregulated. Upregulated genes predominantly belonged to the expansins (EXP), yieldins (GH18), xyloglucan endotransglycosylases/hydrolases (XTH), beta-galactosidases (BGAL), glycoside hydrolases 17 (GH17), pectin acetylesterases (PAE). and glycosyl transferases 21A (GT31a).

### Expression profiles of ROS-related genes

We analyzed the global expression profiles of genes related to 15 ROS-related gene families (Table 4, Additional file 8: Table S4). Among the 343 ROS response-network genes spotted on our arrays, transcripts of 270 showed changed expression after FA treatment (Additional file 8: Table S4): 55 were significantly regulated, 51 upregulated and four downregulated. The genes included alternative oxidases (AOX), glutathione peroxidase (GPx), glutathione reductase (GR), glutaredoxins (Grx), glutathione-S-transferases (GST), monodehydroascorbate reductase (MDAR), class III peroxidase (Prx), peroxiredoxin (PrxR), respiratory burst oxidase homolog (Rboh; NADPH oxidase), and thioredoxin (Trx). Almost all AOX genes were induced by FA, and 25 of the 79 GST genes were significantly upregulated by FA.

**Table 4:** Ferulic acid-responsive transcripts related to ROS

				**Short exposures**		**Long exposures**	
**Functional categories**	**In genome**	**On arrary**	**Detected**	**Increased **^**a**^	**Decreased**	**Increased**	**Decreased**
Reactive oxygen species (ROS) network	343	323	270	51	4	5	2
AOX (Alternative oxidases) genes	4	4	4	4*	0	0	0
APx (Ascorbate peroxidase) genes	11	11	8	0	0	0	0
Cat (Catalase) genes	3	3	3	0	0	0	0
DiOx (Alpha-dioxygenase)	1	1	1	0	0	0	0
Ferritin genes	2	2	2	0	0	0	0
GPx(Glutathione peroxidase) genes	5	5	5	1	0	0	0
GR (Glutathione reductase) genes	3	3	3	2	0	0	0
Grx (Glutaredoxins) genes	27	22	17	5	0	0	0
GST (Glutathione-S-transferases) genes	79	74	67	25*	0	3	2
MDAR (monodehydroascorbate reductase) genes	15	14	8	2	0	0	0
Prx (Class III Peroxidase) genes	138	130	103	7*	4	2	0
PrxR(Peroxiredoxin) genes	8	8	7	2	0	0	0
Rboh (Respiratory burst oxidase homolog; NADPH oxidase) genes	9	9	7	1	0	0	0
SOD (superoxide dismutase) genes	8	8	8	0	0	0	0
Trx (thioredoxin) genes	30	29	27	2	0	0	0

^a^ Functional categories of genes, total number of genes found within the rice genome, numbers of genes present on and detected on arrays, and numbers of genes showing significant differences (FDR <0.1) in transcript abundance are shown in rows and columns labeled accordingly.

ROS families that are overrepresented in the response group are shown with asterisks (P < 0.05).

### Expression profiles of transporter genes

In the rice genome, transporter families are grouped by mode of transport and energy-coupling mechanism into four types: ATP-dependent transporters, secondary transporters, ion channels, and unclassified transporters with unknown mechanisms of action. Among 1,286 transporter-related genes, 1,113 were present on our arrays, and 64 were significantly upregulated with FA treatment (Table 5, Additional file 9: Table S5). Nearly all of the transporters responding to FA were ATP-dependent and secondary transporters. Transporters with changed expression were 17 of the 130 ATP-binding cassette (ABC) transporters and three of the P-type ATPase (P-ATPase) transporters. The major facilitator superfamily (MFS) is the largest family of secondary transporters in the rice genome. Ferulic-acid treatment upregulated nine MFS genes and downregulated two. Transcripts for five proton-dependent oligopeptide transporter (POT) genes and five amino acid/auxin permease (AAAP) genes were upregulated. In addition, four of 123 drug/metabolite transporter (DMT) genes belonging to secondary transporters were upregulated by FA treatment.

**Table 5:** Ferulic acid-responsive transcripts related to transporter

				**Short exposures**		**Long exposures**	
**Family name**	**In genome**	**On array**	**Detected**	**Increase **^**a**^	**Decrease**	**Increase**	**Decrease**
**ATP-dependent**							
ATP-binding Cassette (ABC) Superfamily	130	115	80	17*	1	3	0
P-type ATPase (P-ATPase) Superfamily	45	42	37	3	0	0	0
**Ion channels**							
Ammonia Transporter Channel (Amt) Family	12	8	7	0	2	0	0
Annexin (Annexin) Family	9	6	6	1	0	1	0
Glutamate-gated Ion Channel (GIC) Family of Neurotransmitter	21	12	10	1	0	1	0
Major Intrinsic Protein (MIP) Family	37	33	24	0	0	0	1
**Secondary transporter**							
Amino Acid/Auxin Permease (AAAP) Family	63	52	41	5	0	0	0
Auxin Efflux Carrier (AEC) Family	19	16	11	2	1	0	0
Amino Acid-Polyamine-Organocation (APC) Family	27	22	20	1	0	0	0
Aromatic Acid Exporter (ArAE) Family	14	13	6	1	0	1	0
Arsenite-Antimonite (ArsB) Efflux Family	3	3	3	1	0	0	0
Ca2+:Cation Antiporter (CaCA) Family	16	15	13	1	0	0	0
Chloride Carrier/Channel (ClC) Family	9	9	8	0	1	0	0
Divalent Anion:Na + Symporter (DASS) Family	7	7	5	1	0	0	0
Drug/Metabolite Transporter (DMT) Superfamily	123	106	86	4	1	0	1
K + Transporter (Trk) Family	7	7	2	1	0	0	0
Mitochondrial Carrier (MC) Family	61	59	55	2	0	0	0
Major Facilitator Superfamily (MFS)	151	133	103	9	2	1	1
Multidrug/Oligosaccharidyl-lipid/Polysaccharide (MOP) Flippase Superfamily	57	44	34	5	1	0	0
Monovalent Cation:Proton Antiporter-2 (CPA2) Family	20	18	4	1	0	1	0
Proton-dependent Oligopeptide Transporter (POT) Family	86	74	50	5	0	0	0
Telurite-resistance/Dicarboxylate Transporter (TDT) Family	9	8	4	0	1	1	0
Sulfate Permease (SulP) Family	14	14	13	1	0	0	0
Zinc (Zn^2+^)-Iron (Fe^2+^) Permease (ZIP) Family	18	16	12	2	0	0	0

^a^ Functional categories of genes, total number of genes found within the rice genome, numbers of genes present on and detected on arrays, and numbers of genes showing significant differences (FDR <0.1) in transcript abundance are shown in rows and columns labeled accordingly. Transporter families that are overrepresented in the response group are shown with asterisks (P < 0.05).

These observations were further supported by comparison of metabolism genes by use of MapMan. Genes encoding ATP-binding cassette-type and AAAP transporters were differentially regulated in the early (1 and 3 h) response to FA (Figure 2B). MapMan analysis revealed that AAAP transporters were significantly upregulated by FA treatment.

### Expression profiles of phytohormone-related genes

Among 324 phytohormone-related genes, 297 were present on our arrays, and 25 were significantly upregulated with FA treatment (Table 6, Additional file 10: Table S6). One jasmonic acid (JA) biosynthesis gene, *OsAOS2* (Os03g0767000) and six JA signaling genes (Os03g0180900, Os10g0392400, Os03g0402800, Os03g0181100, Os03g0180800, and Os09g0439200) were upregulated by FA exposure; none were downregulated during the same time of exposure. MapMan analysis revealed that ethylene (ET) synthesis and signaling genes were significantly upregulated by FA treatment (Figure 2C).

**Table 6:** Ferulic acid-responsive transcripts related to phytohormones

					**Short exposures**		**Long exposures**	
**Functional categories**		**In genome**	**On arrary**	**Detected**	**Increased **^**a**^	**Decreased**	**Increased**	**Decreased**
**Ethylene**	**Total**	**29**	**27**	**22**	**3**	**0**	**0**	**0**
	Biosynthesis	13	11	10	3	0	0	0
	Signaling	16	16	12	0	0	0	0
**JA**	**Total**	**38**	**34**	**34**	7*	**0**	**0**	**0**
	Biosynthesis	27	24	16	1	0	0	0
	Signaling	11	10	10	6	0	0	0

^a^ Functional categories of genes, total number of genes found within the rice genome, numbers of genes present on and detected on arrays, and numbers of genes showing significant differences (FDR <0.1) in transcript abundance are shown in rows and columns labeled accordingly.

Phytohormone families that are overrepresented in the response group are shown with asterisks.

### Expression profiles of signaling genes and TFs

**Figure 3:**
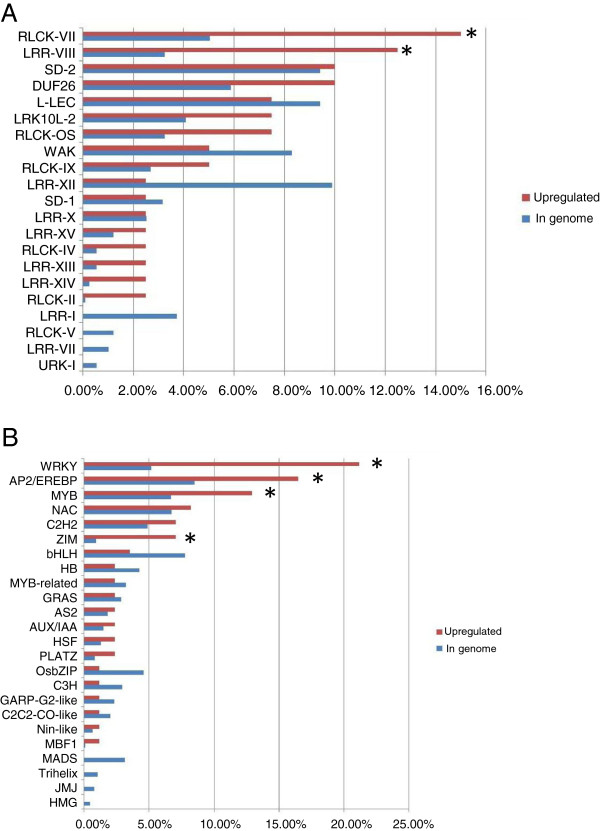
**Family classifications of FA stress response genes.** Twenty-one receptor like kinase (RLK) protein kinase families (**A**) and 24 transcription factor families (**B**) were regulated by FA stress. Red bars represent the percentage of upregulated genes (fold change ≥2; FDR < 0.1) in each protein kinase or transcription factor family. Blue bars refer to the percentage of genes per kinase or transcription factor categories with respect to the entire number of kinases or transcription factors in the genome. Fisher’s exact test was used to assess the significance of overrepresented protein kinase and transcription factor families. Asterisks indicate families that were significantly overrepresented in the response group (*P* < 0.05).

Perception and transmission of stress signals are important aspects of the plant response to environment stress. Protein kinases are crucial in these signaling pathways. The activation of signal transduction pathways connects the actions of protein kinases, TFs and the downstream stress-responsive genes. In total, 51 protein kinase genes were upregulated by FA, and 16 were downregulated (Figure 3A, Additional file 11: Table S7). Nearly all of the FA-responsive kinases were associated with the receptor-like kinase (RLK) family. In total, 40 RLK family genes were significantly upregulated and 15 were downregulated after short and long FA exposure. The leucine-rich repeat VIII (LRR-VIII) and receptor-like cytoplasmic kinases VII (RLCK-VII) subfamilies of the RLK family were significantly upregulated with FA treatment.

We found 107 TFs significantly regulated by FA: 85 were significantly upregulated and 22 downregulated after short and long exposure. Transcription factors regulated by FA stress predominantly belong to the APETALA2/ET response factor (AP2/ERF), MYB, WRKY and Zinc-finger protein expressed in inflorescence meristem (ZIM) families (Figure 3B, Additional file 12: Table S8). From rice genome sequence data, 164, 129, 100 and 18 genes have been identified for the AP2/ERF, MYB, WRKY and ZIM families, respectively. In our rice roots, FA induced 14 AP2/ERF, 11 MYB, 17 WRKY and 6 ZIM families.

### Transporters, TFs, and protein kinases specifically altered by FA and juglone

**Figure 4:**
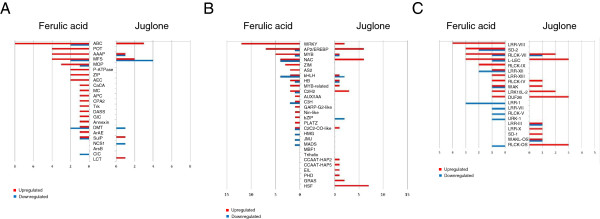
**Comparison of gene regulation by FA and juglone.** FA and juglone specific regulation of transporters (**A**), transcription factor**s** (**B**), and protein kinases (**C**). Genes repressed or activated by FA or juglone are in blue (fold change ≤0.5) and red (fold change ≥2), respectively.

We compared transporters, TFs, and protein kinases regulated by exposure to FA and to the ROS-generating allelochemical juglone (Figure 4). Genes encoding AAAP transporters responded relatively specifically to FA (Additional file 13: Table S9). Comparison of the TFs induced by juglone after short FA exposure revealed that only half of the genes (48 of 84) reported in our previous study [35] showed changed expression in this study (Additional file 14: Table S10). The WRKY and Myb TFs responded significantly to FA stress. Comparison of the protein kinase genes induced by juglone revealed that the LRR-VIII and SD-2b families responded significantly to FA stress (Additional file 15: Table S11).

## Discussion

Autotoxicity is intraspecific allelopathy and plays an important role in plant growth inhibition and structuring plant communities [1]. Here, we observed that treatment with 50 ppm of the autotoxic chemical FA inhibited rice root length by 50% (Figure 1). Plant growth as well as response to stress is controlled by phytohormones [38]. Our microarray assay revealed changed expression of ET and JA biosynthesis and signaling genes in rice roots with FA treatment (Table 6). Xu et al. [39] reported that the combination of ET and JA synergistically induced the expression of defense genes in plants. Hua and Meyerowitz [40] and Staswick et al. [41] reported that JA and ET treatment inhibited plant root elongation. Our results suggest that these two hormones may be involved in FA-induced inhibition of root growth in rice. In addition, we found that FA inhibited crown root, lateral root and root hair formation. FA repressed the expression of two lateral-root genes (*ARF-16*, Os06g0196700; *OsCel9C*, Os05g0212300) and one root-hair–related gene in rice roots (*OsCSLD1*, Os10g0578200) (Table 3). Examination of the RiceXPro database revealed that JA repressed the expression of these genes (*ARF-16*, *OsCel9C* and *OsCSLD1*) [42]. Therefore, our results suggest that JA may involve in FA-induced morphogenic response in roots by regulating these root architecture- related genes.

In general, cell walls become lignified when cell expansion decreases or when the cell is under stress [43]. Previous study indicated that lignification may be an important step in root growth reduction in FA-stressed soybean [21]. Our FA treatment upregulated genes involved in the cell-wall macromolecule metabolic process (GO:0044036, FDR 6.10E-13), particularly cell-wall reassembly. The expression of cell-wall-related genes, most notably the expansins, was enriched by FA treatment. Cosgrove found that expansins are a group of wall proteins that induce wall stress relaxation and extension [44]. Increased expansin gene activity may be involved in stress relaxation in FA-treated roots.

Increased ROS levels are an important component of the stress induced by allelochemicals [45]. Ferulic acid modifies various oxidative reactions *in vitro* by acting as a substrate, activator or inhibitor depending on the concentration [46,47]. Reactive oxygen species are toxic to plant tissues and can trigger cell growth inhibition and cell death. In addition, they may act as signal molecules involved in triggering tolerance against various environmental stresses. In this study, FA rapidly induced ROS production in rice roots. Ferulic acid-induced lipid peroxidation of roots was positively confirmed by Schiff’s reagent staining (Additional file 3: Figure S3). We found a steady increase in LOX activity in response to FA (Additional file 3: Figure S3). Thus, FA may increase ROS accumulation, lipid peroxidation, and LOX activity to affect cell integrity in rice roots and contribute to FA-induced root growth inhibition.

Many studies have provided evidence that ROS signaling is integrated with calcium signaling networks in plants. Saijo et al. [48] and Martín and Busconi [49] demonstrated rapid increase in cytoplasmic calcium concentrations in plant cells in response to multiple stress stimuli. The change in cytoplasmic calcium concentrations is critical for activating various defense responses [50]. We found that FA increased calcium levels in rice roots. Thus, ROS and calcium may act as early second messengers in the transcriptional activation of an array of defense-related genes in rice roots under FA stress.

Conjugated forms of xenobiotics can be recognized by specific membrane-associated transporters in the final detoxification phase [32]. Our GO analysis notably revealed the term “primary active transmembrane transporter activity”. We found 64 membrane-transporter–like sequences induced by FA, including 17 putative ABC, nine MFS, and five AAAP transporters. In plants, ABC and MFS transporters represent different multidrug efflux protein superfamilies associated with resistance to xenobiotics [32]. The ABC transporters facilitate the movement of glutathionylated toxins and other substrates across biological membranes [51]. We found 17 and three ABC transporter genes upregulated by short and long FA exposure, respectively. Thus, expression of ABC transporters, which work in conjunction with other detoxifying systems, was found primarily with early stages of FA stress. The AAAPs are efficient transporters of proline and betaine [52] that accumulate in higher plants under stress conditions such as drought, salinity, extreme temperatures, UV radiation, and heavy metals [53,54]. Previous reports have demonstrated a positive relationship between proline and betaine accumulation and plant stress tolerance [55,56]. Our observed induction of AAAPs by FA indicates their possible involvement in plant tolerance to autotoxin stress.

Protein kinases are important signaling molecules in the plant response to environment stress. Multiple plant RLK members are involved in the stress response [57-59]. Among 40 RLK genes we found upregulated with FA treatment, LRR-VIII and RLCK-VII subfamilies were identified as significantly participating in transcriptional regulation (Additional file 11: Table S7). The involvement of LRR-VIII and RLCK-VII in stress responses was previously reported [58]. Thus, differential expression of a number of transmembrane receptor kinases with FA exposure suggests that multiple receptors belonging to different families may have unique regulatory mechanisms.

Responses to abiotic stresses require the production of important regulatory proteins such as TFs to mediate the expression of downstream stress-responsive genes. We found that the major TFs, AP2/ERF, MYB, WRKY, and ZIM, were overrepresented in the response to FA. The AP2/ERF, MYB, and WRKY TFs have been isolated from different plants and are important candidates for the stress tolerance response; in rice, the overexpression of AP2/ERF, MYB, and WRKY conferred significant tolerance to abiotic stresses [60-63]. Transcription factors of ZIM have been intensively investigated because of the role of these proteins as key regulators of the jasmonate hormonal response in *Arabidopsis* and rice [64]. Here, we found that FA upregulated six ZIM genes. Overexpression of *ZIM-3* (Os03g0180800), a stress-inducible gene, was found to significantly increase tolerance to salt and dehydration stresses [64]. The observed induction of AP2/ERF, MYB, WRKY, and ZIM TFs during FA treatment indicates their possible involvement in plant resistance to autotoxin stress.

Reactive oxygen species are secondary messengers for the activation of specific TFs. We found that FA induced ROS production. Therefore, we compared the set of our FA-regulated TFs to those regulated by exposure to juglone, an ROS-generating allelochemical [35]. Our results suggest that WRKY and Myb TFs and LRR-VIII and SD-2b kinases might regulate downstream genes under FA stress but not general allelochemical stress (Figure 4). Moreover, 64 transporters were upregulated by FA, but only 31 transporters were upregulated by juglone. The number of upregulated genes encoding transporters was more under FA than juglone stress. Especially, the AAAP transporter family was regulated significantly by FA stress but not by juglone (Figure 4). The AAAPs are efficient transporters of osmoprotectants such as proline, glycinebetaine and gamma-aminobutyric acid [52] that accumulate in higher plants under stress conditions. This observation could be related to detoxification of the autotoxin in rice roots. The AAAP transporters may play an important role in the FA-triggered autotoxicity mechanism.

## Conclusions

**Figure 5:**
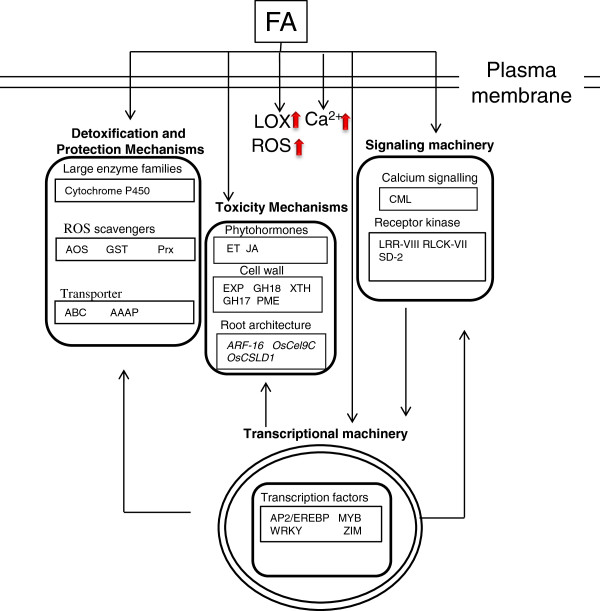
Molecular mode of action of the allelochemical FA in cellular processes and response/regulatory pathways.

FA may have a significant effect on inhibiting rice root elongation through ET and JA gene regulation. Detoxification enzymes such as cytochrome, GST, and ROS scavengers are involved in protecting against FA toxicity. Moreover, proteins involved in regulatory functions and signal transduction, including TFs, calcium-regulated proteins, and various protein kinases, play important roles in the response to FA stress (Figure 5). Future studies with rice mutants or overexpressors with altered expression of the genes identified in this work will be helpful to elucidate their biological significance and clarify new pathways involved in toxicity and tolerance to FA.

## Methods

### Plant materials

Rice plants (*O. sativa* L. cv. TN-67) were grown as previously described [65]. Rice seedlings were exposed to FA (25 to 200 ppm) for 1 to 24 h. Control plants were treated with water in parallel for the indicated times.

### Analysis of growth

Rice seeds were surface-disinfected with 2.5% (v/v) sodium hypochlorite (Katayama, Osaka, Japan) for 15 min, then thoroughly washed in distilled water. Seeds were placed in 9-cm Petri dishes containing 20 ml distilled water and left at 37°C in the dark. After 2 d of incubation, uniformly germinated seeds were transferred to Petri dishes with filter paper discs (Advantec, Tokyo) moistened with 10 ml distilled water. Each Petri dish contained 15 germinated seeds grown at 27°C in the dark for 3 d. Once the roots reached 0.2 cm in length, they were used for experiments of exposure to FA (Sigma, St. Louis, MO, USA) under sterile conditions in the same Petri dish. Ferulic acid was added at final concentrations of 0 to 200 ppm for varying treatment durations. Root length was measured after 3 d of incubation at 26°C in darkness. Mean root length was obtained from 15 individual seedlings from at least 3 separate experiments. To determine the number of crown root and lateral roots, the number of all emerged lateral roots on seminal roots was counted by the naked eye. Root samples of 6-day-old rice seedlings were treated with FA for 3 days. The values of crown root and lateral root number represent the mean of 15 seedlings. The value of lateral root length represents the mean of 200 lateral roots. For root hair measurement, after 24-h FA treatment, the number and length of root hairs on the root hair zone immediately behind the root tip (3–4 mm behind the root tip) of seminal roots were determined by microscopy (Leica MZ125) (Leica Microsystems, Heerbrugg, Switzerland). To determine number of root hairs, the number of root hairs from one side of the root hair zone of seminal roots was counted. To determine the length of root hairs, the length of the 20 longest root hairs from the root hair zone of seminal roots was measured.

### Detection of ROS and calcium levels in rice roots

Root samples of 6-day-old rice seedlings were labeled with 10 μM CM-H_2_DCF-DA (Molecular Probes, Eugene, OR, USA) or Oregon Green 488 BAPTA-1 (Molecular Probes) for 30 min to determine ROS or calcium levels, respectively, then treated with 50 ppm FA for 1–3 h. Fluorescence images were visualized under a confocal microscope (EZ-C1; Nikon, Tokyo, Japan) with the 488-nm laser line of an Ar laser (2 mW optical fiber output; 500–530 nm). Exposure times were equal for all samples.

### Purification of total RNA

Total RNA was extracted from rice plants grown as described above [35] and treated with 50 ppm FA for 1–24 h. Roots were separated from shoots, and total RNA was isolated from root tissues with use of the RNeasy Plant Mini kit (QIAGEN, Hilden, Germany). The RNA was further treated with DNase (QIAGEN) to eliminated DNA contamination. The concentrations of total RNA samples were measured with use of NanoDrop ND2000 (NanoDrop Technologies, Wilmington, DE, USA). The purity of RNA samples was determined by OD_260/280_ and OD_260/230_. RNA samples of more than 2 μg/μl concentration and high purity (OD_260/280_ > 2, OD_260/230_ > 2) were used for microarray assay and RT-PCR.

### Microarray preparation and analysis

Six-day-old rice seedlings were exposed to 50 ppm FA for short (1 and 3 h) or long (24 h) durations, then RNA was isolated from root tips to examine rapid changes in global patterns of gene expression. We pooled RNA from the two short exposures to maximize gene discovery. RNA from water-treated (control) and FA-treated roots was used with the Agilent Rice Oligo microarray (4 × 44 K, custom-made; Agilent Technologies, Palo Alto, CA, USA) for RNA labeling and microarray hybridizations involved 3 biological replicate samples.

For the microarray assay, 0.5 μg total RNA was amplified by use of the Fluorescent Linear Amplification Kit (Agilent) and labeled with Cy3-CTP (control samples) or Cy5-CTP (FA-treated) (CyDye, PerkinElmer, Norwalk, CT, USA) during the *in vitro* transcription process. In total, 0.825 μg Cy-labeled cRNA was fragmented to a mean size of about 50–100 nt by incubation with fragmentation buffer (Agilent) at 60°C for 30 min. The fragmented-labeled cRNA was then pooled and hybridized to the Rice Oligo DNA Microarray 44 K RAP-DB (G2519F#15241; Agilent) at 60°C for 17 h. After a washing and blow-drying with a nitrogen gun, microarrays were scanned with use of an Agilent microarray scanner at 535 nm for Cy3 and 625 nm for Cy5. Scanned images were analyzed with use of Feature Extraction v9.5.3 (Agilent), with LOWESS normalization.

Signal intensities were extracted with use of Feature Extraction v9.5.3. For statistical analysis, we excluded genes with signal intensities < 100 in all experiments. Significant differences from 0 were identified by use of *t* test with GeneSpringGX11 (Agilent). The Benjamini-Hochberg false discovery rate (FDR) method was used to obtain *P*-values that were corrected for multiple testing. The fold change in expression of each gene after FA treatment was calculated by the mean from 3 biological replicates. Genes upregulated by FA treatment by more than two-fold (cutoff by FDR < 0.1) were extracted. Each probe was considered an individual gene and annotated according to the Rice Annotation Project Data Base (RAP-DB; http://rapdb.dna.affrc.go.jp/; Rice Annotation Project 2007, 2008). The dye swap was not included. Three biological replicates were performed with 3 independent microarray slides for both short- and long-term FA treatments. Total RNA control samples were labeled with Cy3, and total RNA experimental samples (FA treatment) were labeled with Cy5.

FA-responsive genes were annotated according to the RAP-DB and TIGR Rice Genome Annotation Resource (http://rice.plantbiology.msu.edu/) [66] and were classified into functional categories by AgriGO gene ontology (GO) functional enrichment analysis [67]. For signaling, transcription factor (TF), and peroxidase functions, the lists of rice genes encoding protein kinases (1,467 genes), TFs (1,930 genes), the main ROS (343 genes), cell-wall–related genes (639 genes), and transporters (1,286 genes) were obtained from the Rice Kinase Database (http://rkd.ucdavis.edu) [68], the Database of Rice Transcription Factors (DRTF; http://drtf.cbi.pku.edu.cn/) [69], the peroxidase database (http://peroxibase.toulouse.inra.fr/) [70], Cell Wall Navigator (CWN; http://bioinfo.ucr.edu/projects/Cellwall/index.pl) [71], and TransportDB (http://www.membranetransport.org) [72], respectively. Fisher’s exact test (*P* < 0.05) [73] was used to assess the significance of overrepresented ROS, cell-wall, transporters, protein kinases and TFs in the list of regulated genes in the genome. The microarray data described in this study have been deposited in the Gene Expression Omnibus and are accessible with the series accession number [GEO: GSE34899] (http://www.ncbi.nlm.nih.gov/geo/query/acc.cgi?acc=GSE34899) [74].

### MapMan display

The averaged signals for a given treatment were expressed relative to those for control samples, converted to a log2 scale and displayed by use of MapMan v3.5.1 [75]. *O. sativa* mapping files were imported into MapMan. Rice genes represented on the Rice Oligo DNA Microarray were organized by BINS and sub-BINS for display on the schematic map of the transport overview. Gene expression was analysed by the Wilcoxon Rank Sum test with uncorrected p value.

### Semi-quantitative RT-PCR

Total RNA was isolated from root tissues treated with 50 ppm FA for 3, 12, or 24 h by use of the RNeasy Plant Mini kit (QIAGEN) and purified with the RNase-Free DNase Set (QIAGEN). Primer sequences are in Supporting Information (Additional file 16: Table S12). The number of PCR cycles in the experiments was adjusted to the optimal conditions. The data was shown on the basis of at least three biological replicates. Amplicons were analyzed by 1% agarose gel electrophoresis, and PCR products were sequenced.

### Histochemical analyses and in-gel enzyme analyses

Histochemical detection of lipid peroxidation involved use of Schiff’s reagent [76]. In brief, freshly harvested rice roots were stained with Schiff’s reagent for 60 min, which detects aldehydes originating from lipid peroxides. Then roots were rinsed with potassium sulphite solution (0.5% [w/v] K_2_S_2_O_5_ prepared in 0.05 M HCl) and maintained in the solution. The isozymes of lipoxygenase (LOX) were separated on discontinuous polyacrylamide gels (stacking gel 4.5%, separating gel 10%) under non-denaturing and non-reducing conditions. Proteins were electrophoretically separated at 4°C and 80 V in the stacking gel, then 120 V in the separating gel. Isozymes of LOX were visualized as described [77].

## Abbreviations

AAAP: Amino acid/auxin permease; ABC: ATP-binding cassette; AOX: Alternative oxidases; AP2/ERF: APETALA2/ET response factor; BGAL: Beta-galactosidases; CM-H2DCF-DA: 5-(and-6)-chlormethyl-2′,7′-dichlordihydrofluorescein diacetate, acetyl ester; DCF: Dihydrodichlorofluorescein; DMT: Drug/metabolite transporter; DRTF: The Database of Rice Transcription Factors; ET: Ethylene; EXP: Expansins; FA: Ferulic acid; FDR: False discovery rate; GH17: Glycoside hydrolases 17; GH18: Yieldins; GO: Gene ontology; GPx: Glutathione peroxidase; GR: Glutathione reductase; Grx: Glutaredoxins; GST: Glutathione S-transferases; JA: Jasmonic acid; LOX: Lipoxygenase; LRR-VIII: Leucine-rich repeat VIII; MDAR: Monodehydroascorbate reductase; MFS: Major facilitator superfamily; PAE: Pectin acetylesterases; PAL: Phenylalanine ammonia-lyase; POD: Peroxidase; POT: Proton-dependent oligopeptide transporter; Prx: Class III peroxidase; PrxR: Peroxiredoxin; P-ATPase: P-type ATPase; Rboh: Respiratory burst oxidase homolog; RLCK: Receptor-like cytoplasmic kinase; RLK: Receptor-like kinase; ROS: Reactive oxygen species; TFs: Transcription factors; Trx: Thioredoxin; XTH: Xyloglucan endotransglycosylases/hydrolases; ZIM: Zinc-finger protein expressed in inflorescence meristem

## Competing interests

The authors declare that they have no competing interests.

## Authors’ contributions

WCC and YAC carried out the microarray studies and drafted the manuscript. YCH carried out the RT-PCR. SFF, CHC, NNT and YCC participated in the design of the study. HJH conceived the study, participated in its design and coordination, and helped to draft the manuscript. All authors read and approved the final manuscript.

## Supplementary Material

Additional file 1: Figure S1. Detection of superoxide accumulation in rice roots during ferulic acid (FA) stress with nitroblue tetrazolium (NBT) staining. Rice seedling roots were treated with 50 ppm FA for 1–3 h. Click here for file

Additional file 2: Figure S2 Reactive oxygen species (ROS) production and calcium accumulation in rice roots during FA stress. (**A**) Root samples were labeled with 10 μM CM-H_2_DCF-DA for 30 min and treated with 50 ppm FA for 1–3 h. (**B**) Root samples were labeled with 10 μM Oregon Green 488 BAPTA-1, a calcium indicator, for 30 min and treated with 50 ppm FA for 1–3 h. The signals were quantified by use of ImageJ program producing histograms of signal intensity. The signal of the first sample on the panel was defined as 1.0 (arbitrary units), and other abundances were expressed relative to that value. Intensity values in each panel are color coded to represent the relative fold change in expression. Click here for file

Additional file 3: Figure S3 Lipid peroxidation in rice roots and time course of the response of lipoxygenase (LOX) activity with ferulic acid (FA) treatment in rice roots. (**A**) FA-induced lipid peroxidation. Roots were stained with Schiff’s reagent. (**B**) Rice roots were treated with 50 ppm FA for 3–24 h. Native polyacrylamide gel electrophoresis of root extracts containing 200 μg protein. Click here for file

Additional file 4: Table S1 Genes with expression responding to 50 ppm ferulic acid. Click here for file

Additional file 5: Table S2 Gene ontology analysis of 972 genes upregulated with 50 ppm ferulic acid. Click here for file

Additional file 6: Figure S4 Verification of microarray data by RT-PCR. The number of PCR cycles in the experiments was adjusted to the optimal conditions. The data was shown on the basis of at least three biological replicates. Click here for file

Additional file 7: Table S3 Expression profiles of cell wall-related genes induced by 50 ppm ferulic acid. Click here for file

Additional file 8: Table S4 Expression profiles of ROS-related genes induced by ferulic acid stress. Click here for file

Additional file 9: Table S5 Expression profiles of transporter genes induced by ferulic acid stress. Click here for file

Additional file 10: Table S6 Expression profiles of phytohormone-related genes induced by ferulic acid stress. Click here for file

Additional file 11: Table S7 Expression profiles of protein kinase genes induced by ferulic acid stress. Click here for file

Additional file 12: Table S8 Expression profiles of transcription factors induced by ferulic acid stress. Click here for file

Additional file 13: Table S9 Expression profiles of transporter genes induced by short exposure to ferulic acid or juglone. Click here for file

Additional file 14: Table S10 Expression profiles of transcription factors induced by short exposure to ferulic acid or juglone. Click here for file

Additional file 15: Table S11 Expression profiles of protein kinase genes induced by short exposure to ferulic acid or juglone. Click here for file

Additional file 16: Table S12 Oligonucleotide primers for semi-quantitative RT-PCR. Click here for file
