# Supplementary figures and images for: Autotoxicity mechanism of Oryza sativa: transcriptome response in rice roots exposed to ferulic acid
Source: BMC Genomics. 2013 May 25;14:351. doi: 10.1186/1471-2164-14-351 (PMC4008027; doi:10.1186/1471-2164-14-351)

## Slide 1
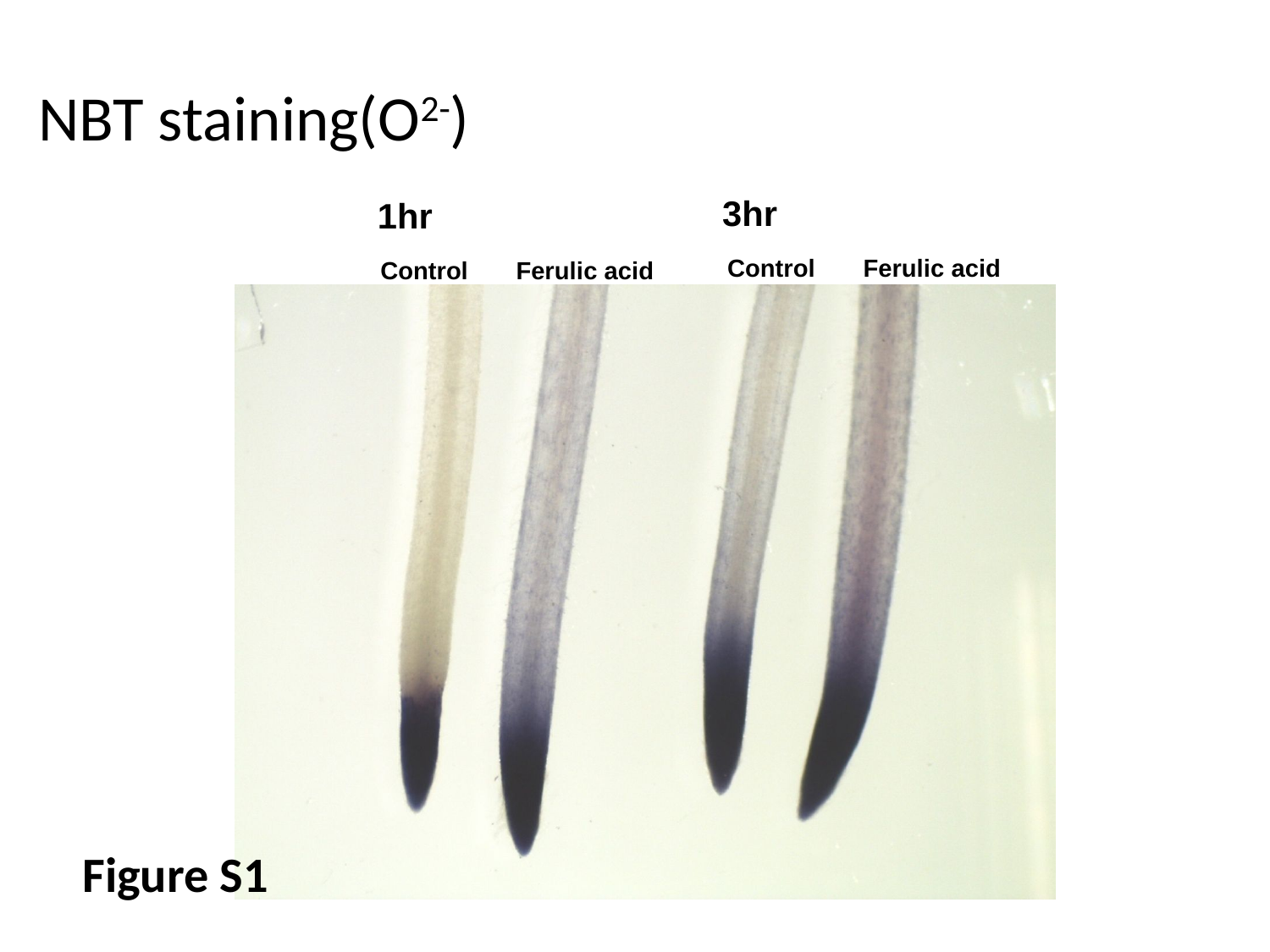

# NBT staining(O2-)
3hr
1hr
Control
Ferulic acid
Control
Ferulic acid
Figure S1

Supplement: Additional file 1: Figure S1. — Detection of superoxide accumulation in rice roots during ferulic acid (FA) stress with nitroblue tetrazolium (NBT) staining. Rice seedling roots were treated with 50 ppm FA for 1–3 h. [file 1471-2164-14-351-S1.ppt]

## Slide 1
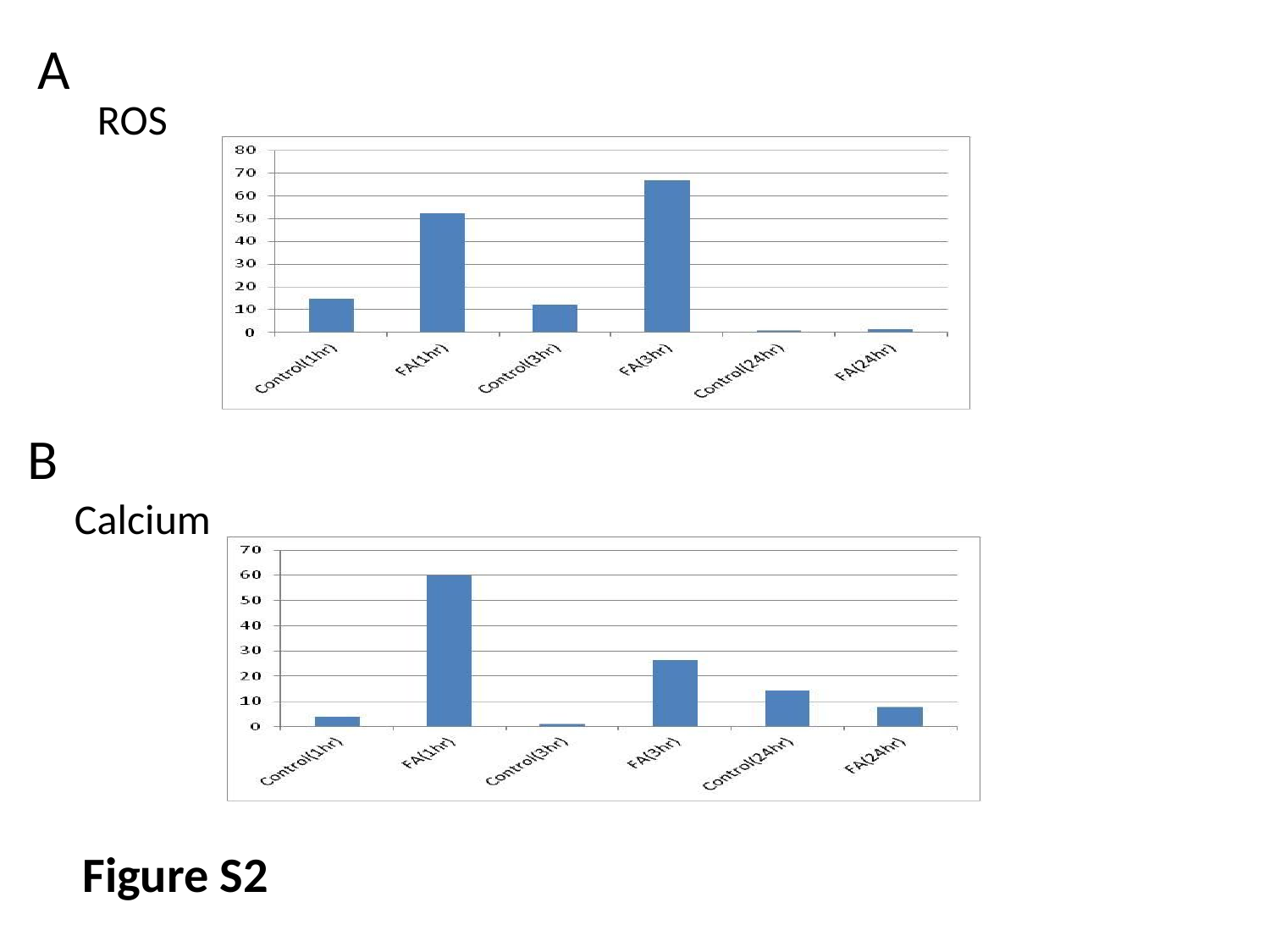

A
ROS
B
Calcium
Figure S2

Supplement: Additional file 2: Figure S2 — Reactive oxygen species (ROS) production and calcium accumulation in rice roots during FA stress. (A) Root samples were labeled with 10 μM CM-H2DCF-DA for 30 min and treated with 50 ppm FA for 1–3 h. (B) Root samples were labeled with 10 μM Oregon Green 488 BAPTA-1, a calcium indicator, for 30 min and treated with 50 ppm FA for 1–3 h. The signals were quantified by use of ImageJ program producing histograms of signal intensity. The signal of the first sample on the panel was defined as 1.0 (arbitrary units), and other abundances were expressed relative to that value. Intensity values in each panel are color coded to represent the relative fold change in expression. [file 1471-2164-14-351-S2.ppt]

## Slide 1
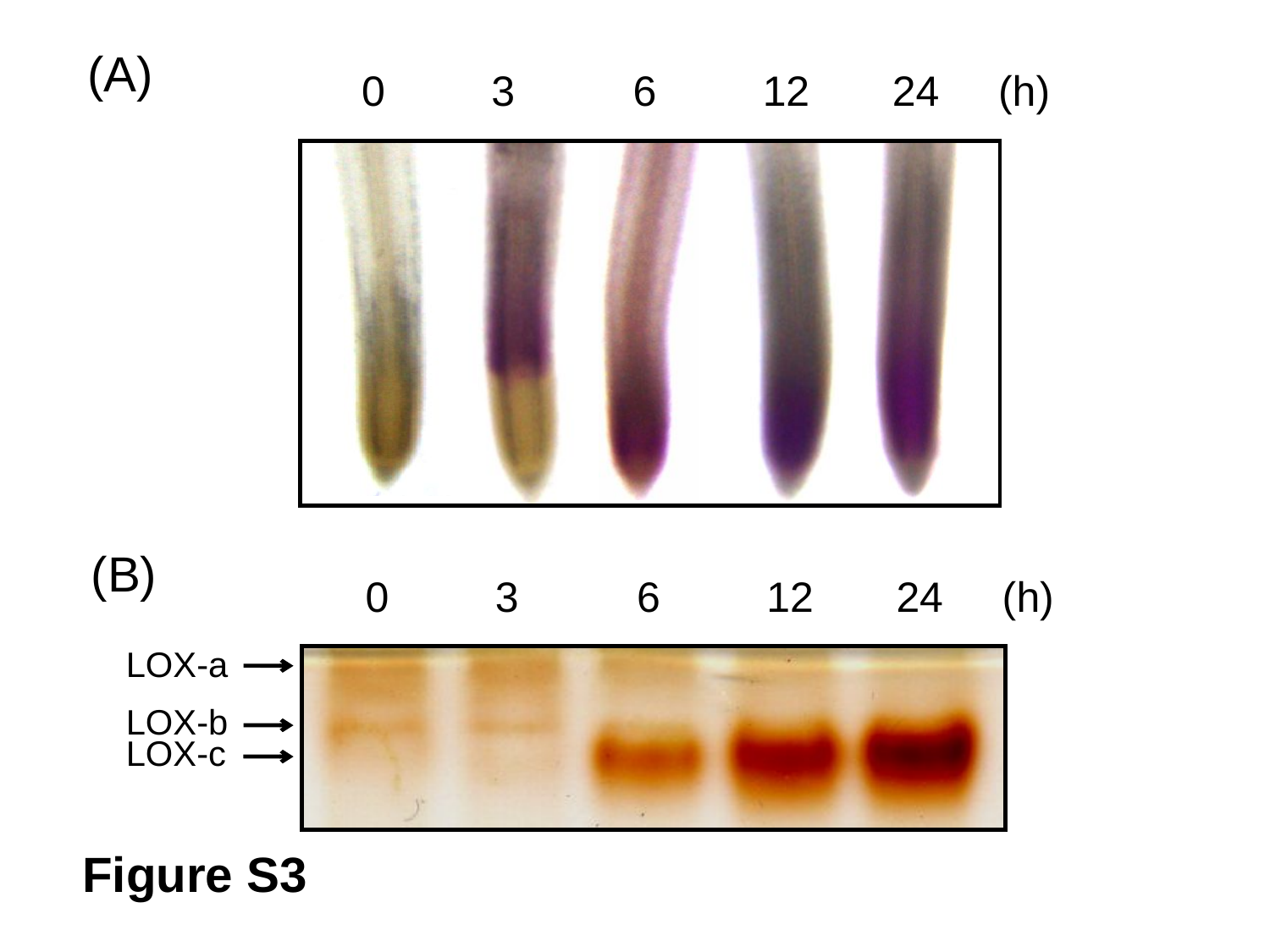

(A)
 0 3 6 12 24 (h)
(B)
 0 3 6 12 24 (h)
LOX-a
LOX-b
LOX-c
Figure S3

Supplement: Additional file 3: Figure S3 — Lipid peroxidation in rice roots and time course of the response of lipoxygenase (LOX) activity with ferulic acid (FA) treatment in rice roots. (A) FA-induced lipid peroxidation. Roots were stained with Schiff’s reagent. (B) Rice roots were treated with 50 ppm FA for 3–24 h. Native polyacrylamide gel electrophoresis of root extracts containing 200 μg protein. [file 1471-2164-14-351-S3.ppt]
