# Supplementary material for: Autotoxicity mechanism of Oryza sativa: transcriptome response in rice roots exposed to ferulic acid
Source: BMC Genomics. 2013 May 25;14:351. doi: 10.1186/1471-2164-14-351 (PMC4008027; doi:10.1186/1471-2164-14-351)
Supplement: Additional file 6: Figure S4 — Verification of microarray data by RT-PCR. The number of PCR cycles in the experiments was adjusted to the optimal conditions. The data was shown on the basis of at least three biological replicates. [file 1471-2164-14-351-S6.ppt]

## Slide 1
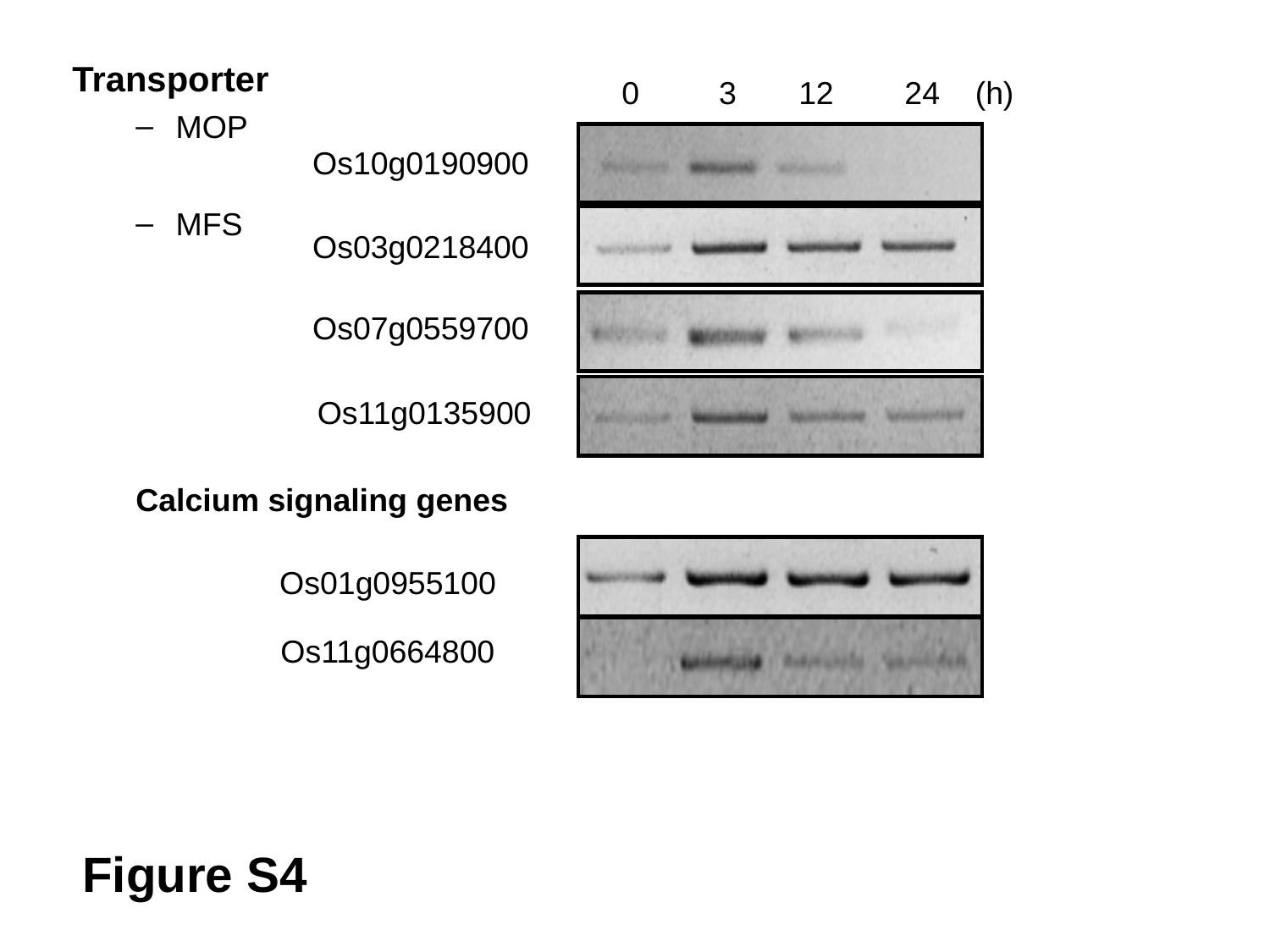

Transporter
MOP
MFS
Calcium signaling genes
 0 3 12 24 (h)
Os10g0190900
Os03g0218400
Os07g0559700
Os11g0135900
Os01g0955100
Os11g0664800
Figure S4

## Slide 2
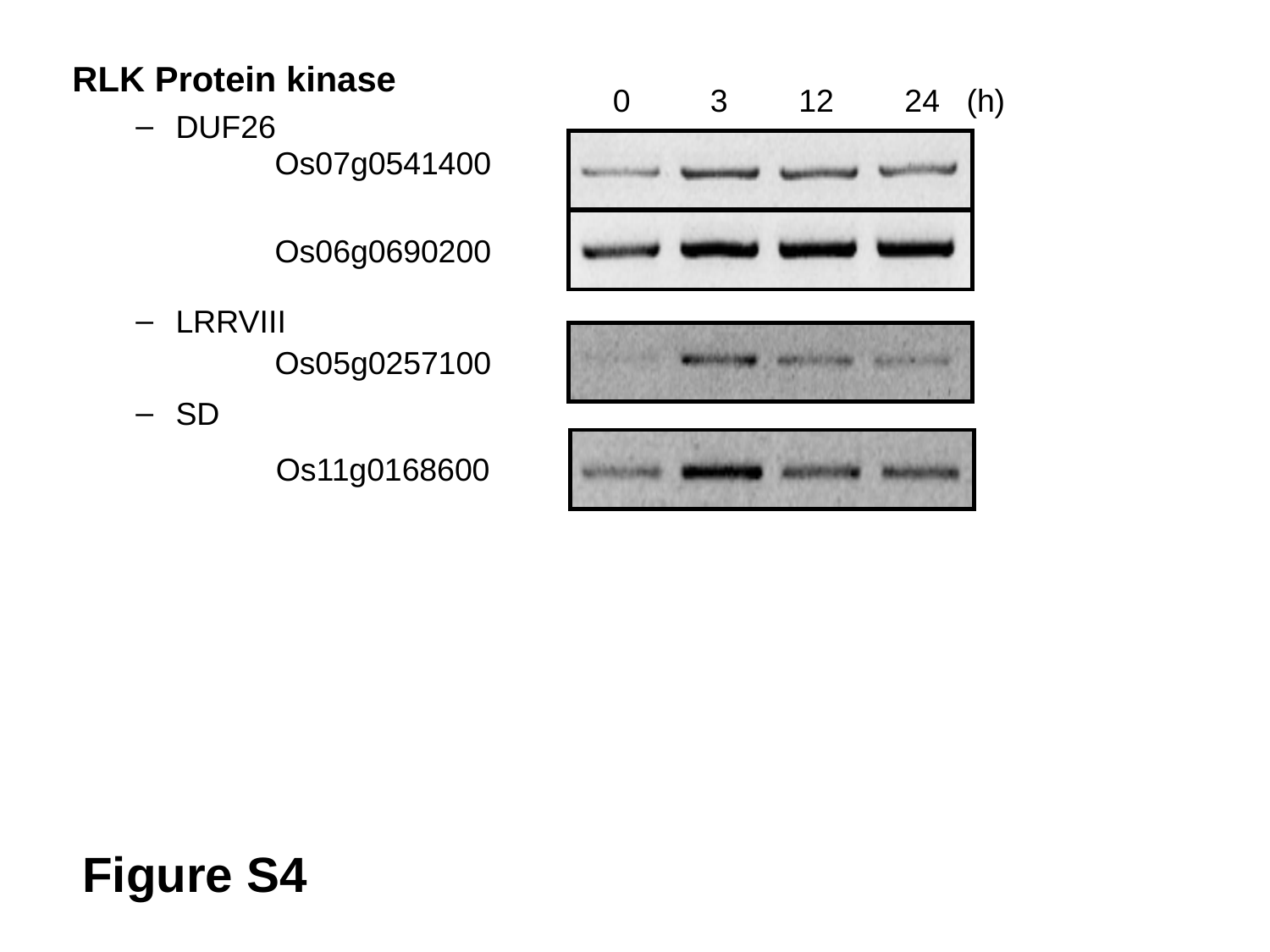

RLK Protein kinase
DUF26
LRRVIII
SD
 0 3 12 24 (h)
Os07g0541400
Os06g0690200
Os05g0257100
Os11g0168600
Figure S4

## Slide 3
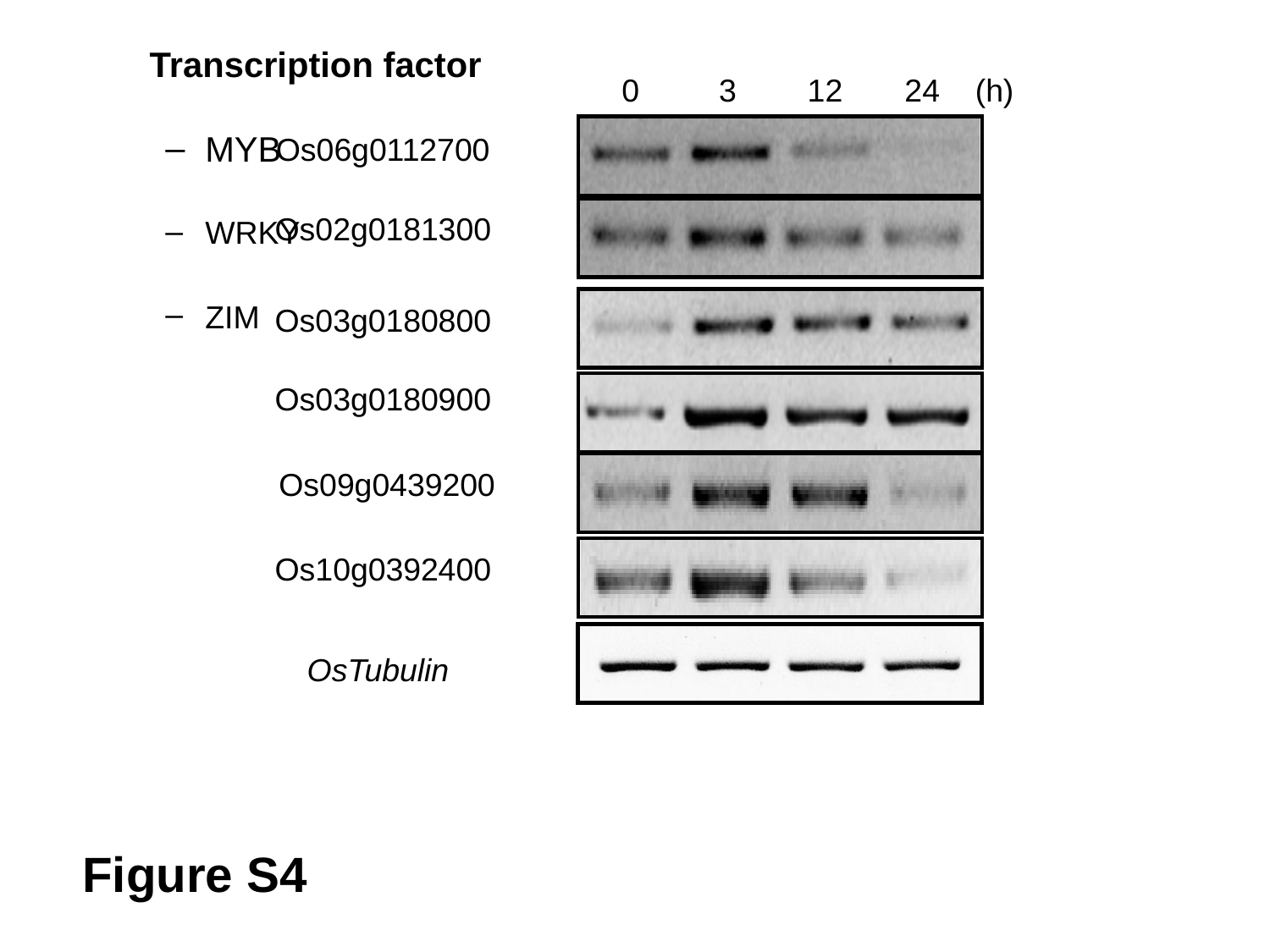

# Transcription factor
MYB
WRKY
ZIM
 0 3 12 24 (h)
Os06g0112700
Os02g0181300
Os03g0180800
Os03g0180900
Os09g0439200
Os10g0392400
OsTubulin
Figure S4
